# Quality intrapartum care expectations and experiences of women in sub-Saharan African Low and Low Middle-Income Countries: a qualitative meta-synthesis

**DOI:** 10.1186/s12884-022-05319-1

**Published:** 2023-01-14

**Authors:** Salma A. E. Ahmed, Abela Mahimbo, Angela Dawson

**Affiliations:** Faculty of Health, University of Technology Sydney, Sydney, Australia

**Keywords:** Woman-centred care, Intrapartum care, Quality of care, Experiences, Expectations, Sub-sahara Africa, Low and low Middle-Income Countries

## Abstract

**Background:**

Woman-centred maternity care is respectful and responsive to women’s needs, values, and preferences. Women’s views and expectations regarding the quality of health services during pregnancy and childbirth vary across settings. Despite the need for context-relevant evidence, to our knowledge, no reviews focus on what women in sub-Saharan African Low and Low Middle-Income Countries (LLMICs) regard as quality intrapartum care that can inform quality guidelines in countries.

**Methods:**

We undertook a qualitative meta-synthesis using a framework synthesis to identify the experiences and expectations of women in sub-Saharan African LLMICs with quality intrapartum care. Following a priori protocol, we searched eight databases for primary articles using keywords. We used Covidence to collate citations, remove duplicates, and screen articles using a priori set inclusion and exclusion criteria. Two authors independently screened first the title and abstracts, and the full texts of the papers. Using a data extraction excel sheet, we extracted first-order and second-order constructs relevant to review objectives. The WHO framework for a positive childbirth experience underpinned data analysis.

**Results:**

Of the 7197 identified citations, 30 articles were included in this review. Women’s needs during the intrapartum period resonate with what women want globally, however, priorities regarding the components of quality care for women and the urgency to intervene differed in this context given the socio-cultural norms and available resources. Women received sub-quality intrapartum care and global standards for woman-centred care were often compromised. They were mistreated verbally and physically. Women experienced poor communication with their care providers and non-consensual care and were rarely involved in decisions concerning their care. Women were denied the companion of choice due to cultural and structural factors.

**Conclusion:**

To improve care seeking and satisfaction with health services, woman-centred care is necessary for a positive childbirth experience. Women must be meaningfully engaged in the design of health services, accountability frameworks, and evaluation of maternal services. Research is needed to set minimum indicators for woman-centred outcomes for low-resource settings along with actionable strategies to enhance the quality of maternity care based on women’s needs and preferences.

**Supplementary Information:**

The online version contains supplementary material available at 10.1186/s12884-022-05319-1.

## Introduction

Many countries have made significant progress toward decreasing maternal mortality; however, much work is required to reach the Sustainable Development Goal (SDG) global target of less than 70 per 100,000 live births by 2030 [1]. Eighty percent of maternal deaths are preventable [2]. An estimated 295,000 maternal deaths occurred globally in 2017 due to pregnancy and delivery-related causes [1]. Despite the substantial progress of countries toward increasing access to maternity services, this has not been reflected in decreasing maternal mortality and morbidity as much as expected [3]. This mismatch between health outcomes and access to services is attributed to the poor quality of services provided to women during pregnancy, childbirth, and postpartum periods [3, 4]. The reduction in maternal and neonatal deaths requires a rapid improvement in the quality and coverage of health services in low and low-middle-income countries (LLMICs).

A fundamental strategy for reducing maternal mortality is increasing access to skilled attendance during childbirth. Skilled birth attendance involves trained, competent, and motivated health workers delivering evidence-based interventions in an enabling environment [5]. A skilled birth attendant (SBA) is a care provider, often a nurse, a midwife, or a doctor, trained to manage normal delivery, detect danger signs, and refer women in a timely manner to receive specialized care [6]. An enabling environment involves the presence of essential medicines and equipment, alongside a functioning referral system [6]. Globally, around 80% of births are assisted by a skilled attendant [7]. However, the coverage of skilled birth attendance varies within countries and across regions. 77% of births are attended by an SBA in Central and Southern Asia while around 59% of births are attended by a skilled provider in Sub-Saharan Africa [7].

While women are encouraged to give birth to their babies with the assistance of a skilled provider in a health facility, facilities may be understaffed, overcrowded, and provide low-quality services [8]. A systematic review of factors affecting the provision of maternal services in LLMICs has shown that lack of supportive supervision, understaffing, and high workloads of care providers contribute to the decreased quality of services [9]. Moreover, low salaries and poor working conditions also contribute to provider stress and performance alongside a lack of equipment and medicines [9]. The initiatives to increase the coverage of skilled birth attendance must go hand in hand with strategies to guarantee that women receive quality services before, during, and after childbirth.

Woman-centred maternity care is defined as respectful care that is responsive to women’s needs, values, and preferences [10, 11]. In 2018, the World Health Organization (WHO) published a set of recommendations for a positive experience during pregnancy and childbirth as part of their support for global high-quality antenatal, delivery, and postnatal care [12]. These recommendations embrace the optimization of the health and well-being of women and their babies through a woman-centred approach rather than a focus on the prevention of mortality and morbidity during pregnancy. The dimensions of the WHO intrapartum care model for a positive childbirth experience include respectful maternity care, emotional support during childbirth, effective communication, pain management, continuity of care, regular monitoring during childbirth, skills, competency and practice of skilled birth attendants, and the physical environment during childbirth [13]. In this model, the WHO describes intrapartum care as.

*A platform to provide pregnant women with respectful, individualised, woman-centred, and effective clinical and non-clinical practices to optimise birth outcomes for the woman and her baby, by skilled healthcare providers in a well-functioning healthcare system* [12].

A systematic review explored women’s needs during childbirth globally in 2018, however, the majority of studies included in this review were conducted in high and middle-income countries, and it only included three studies from African Sub-Saharan (SSA) LLMICs [14]. To our knowledge, no systematic review has been published that focuses on the expectations of women in SSA LLMICs and what women regard as quality care during childbirth. The perspectives of women on what matters to them will support the evidence base for the contextualisation and operationalisation of WHO guidelines on intrapartum care for a positive childbirth experience in SSA LLMICs. The findings can inform the planning, implementation, and appraisal of maternity services which includes the development of woman-centred policies and service guidelines.

## Methods

This qualitative meta-synthesis was conducted following a priori protocol registered on The International Prospective Register of Systematic Reviews (PROSPERO) (Ref. CRD42021292682). Qualitative meta-synthesis is a structured approach to summarising, collating, and interpreting primary qualitative data and the interpretations reported in peer-reviewed articles [15]. A preliminary literature search was undertaken prior to the development of the protocol to refine the review question, determine the feasibility of the review and the nature of current evidence, and decide the synthesis method. The review question was “What are women’s experiences and expectations of quality intrapartum care in SSA LLMICs”. We designed and reported this review in accordance with the Preferred Reporting Items for Systematic Reviews and Meta-Analyses (PRISMA) guidelines [16].

### Search strategy and study selection

**Fig. 1:**
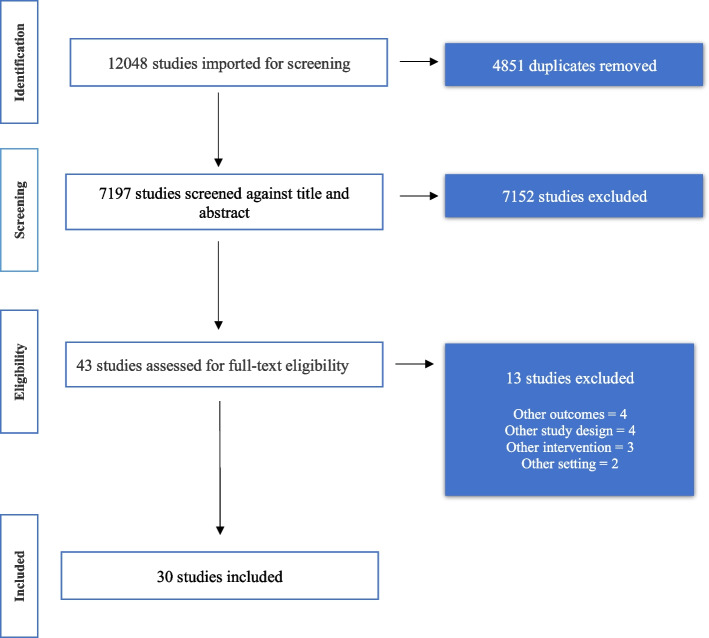
Screening and selection process

We developed a search strategy for qualitative and mixed-methods peer-reviewed articles published between 2011 and 2021. The electronic search was run using eight databases including MEDLINE (Ovid), Global Health, EMBASE (Ovid), CINAHL Plus, Web of Science, SCOPUS, Africa Journals Online (AJOL), and the Maternity and Infant Care Index. The search terms included three main concepts; expectations or satisfaction of women, quality intrapartum care, and eligible geographical scope and countries. Please refer to Supplementary file 1 for a sample of the search strategies used for MEDLINE (Ovid). All searches were conducted from the 13th of December 2021 to the 16th of December 2021. All citations retrieved from electronic searches in databases were imported into a web-based software platform, Covidence, which is used for the management of collation of citations, removal of duplicates, and screening processes. The first author carried out this phase. Two authors (SAEA, AM) independently screened the title and abstracts of identified citations using Covidence. Furthermore, two authors (SAEA, AM) screened full texts for eligibility using inclusion and exclusion criteria (Supplementary file 2) for the final included studies selected (*n* = 30) (Fig. 1). Disagreements during screening were resolved through having a third opinion (AD) and discussions with the research team. The searches of the targeted databases yielded 7197 citations. After the screening of title and abstracts, full texts of potential eligible 43 articles were retrieved. After exclusions, 30 articles were included in this systematic review (Fig. 1).

### Quality appraisal

Two reviewers independently appraised included studies using a quality appraisal checklist. We used the Critical Appraisal Skills Programme (CASP) checklist to assess the quality of peer-reviewed qualitative studies (Supplementary files 3 and 4) [17]. Whereas, mixed methods peer-reviewed articles, we used the Mixed Methods Appraisal Tool (MMAT) (Supplementary file 5) [18]. Disagreements during quality appraisal were resolved by discussion of the team. Quality appraisal tools were used to highlight the strengths and weaknesses of studies to assist in the interpretation of the findings. No studies were excluded during the quality appraisal process.

### Data extraction, analysis, and synthesis

We conducted a framework synthesis, which involves the identification of preliminary themes based on existing framework/s [19, 20]. Data relevant to review objectives were extracted and summarised by the first author using a data extraction excel sheet. This sheet included first-order constructs i.e. women’s views and accounts illustrated in quotes reported in primary articles and second-order constructs i.e. primary studies’ authors’ interpretations of women’s perspectives. The first author developed the data extraction sheet for the purpose of this review. It included a priori list of themes adopted from the components of the WHO framework for a positive childbirth experience to capture women’s perspectives on quality care. The sheet was piloted in the extraction of data from three articles before use. The variables included the article’s characteristics including the aims, number and demographic features of the study population, the study context, and key areas of a positive birth experience. These areas were feeling welcomed, timeliness of care, perceived appropriateness of care, dignified care and respect, access to emotional support during childbirth, patient-provider communication and engagement, continuity of care, pain management, and responsive healthcare providers and physical environment during childbirth. The sheet described the elements included in each theme for consistency. These themes formed a priori framework for the synthesis. We adopted a deductive approach for the extraction and coding of data based on these pre-existing themes, while thematic analysis was used for data that could not be categorised under these themes using an inductive approach (Table 1). Examples of themes/codes that emerged from data include the acceptability of mistreatment and the infrastructural factors behind the denial of a birth companion. The a priori framework was revised to accommodate new codes by reorganising themes. We conducted a framework synthesis of evidence to generate context-specific interpretations and policy-oriented recommendations. For the first stage, we used first and second-order constructs. In contrast, for the synthesis, we used first, second and third-order constructs, which illustrate the views and interpretations of the review team of first and second-order constructs.

**Table 1:** Themes reported in primary articles

Source	Dignified care and respect	Communication and meaningful engagement in care	Access to emotional support during childbirth	Continuity of care	pain management	Responsiveness
Afulani et al.(2017) [21]	Importance of the positive attitude of staff for a positive childbirth experience Negative experiences related to dignified care and respect	Negative experiences related to communication and engagement in care Women’s needs related to communication	Significance of emotional and physical support from HCPs for a positive childbirth experience		Negative experiences related to pain management	Significance of physical environment in having a positive experience Women’s needs regarding responsiveness and physical environment Negative experiences related to responsiveness
Kumbani et al. (2012) [22]	Positive and negative experiences related to dignified care and respect	Negative experiences related to communication and engagement in care			Negative experiences related to pain management	Significance of feeling welcomed Negative experiences related to responsiveness
Jolly et al. (2019) [23]	Expectations of women regarding respectful care Importance of non-judgmental HCPs for a positive childbirth experience	Women’s ability to make informed decisions Women’s needs related to communication during childbirth				Significance of support around the clock and timeliness of care
O’Donnell et al. (2014) [24]	Negative experiences related to respectful care	Negative experiences related to communication and engagement in care	Positive experiences related to access to emotional support			Significance of feeling welcomed and timeliness of care for positive experience
Maya et al. (2018) [25]	Negative experiences related to respectful care Expectations of women regarding respectful care					Significance of feeling welcomed for positive experience Negative experiences related to responsiveness
Dalinjong et al. (2018) [26]	Negative experiences related to respectful care		Positive experiences related to access to support			
Namujju et al. (2018) [27]	Negative experiences related to respectful care	Negative experiences related to communication and engagement in care	Roles of birth companion in provision of support during labor Preferences of women regarding birth companions		Perceptions of women related to labor pain Negative experiences related to pain management	Negative experiences related to responsiveness
Afulani et al. (2018) [28]			Reasons why women need a birth companion Positive and negative experiences related to birth companion			
Bohren et al. (2017) [30]	Negative experiences related to respectful care Expectations of women regarding respectful care	Expectations of women regarding communication	Expectations of women related to access to emotional support	Expectations of women regarding continuity of care		Women’s needs regarding physical environment
Kyaddondo et al. (2017) [32]	Women expectations regarding respectful care Negative experiences related to respectful care	Significance of communication for a positive childbirth experience Negative experiences related to communication and engagement in care	Women’s needs related to emotional support during childbirth	Expectations of women regarding continuity of care		Women’s needs regarding physical environment Significance of feeling welcomed for a positive experience
Mehretie Adinew and Abera Assefa (2017) [33]	Negative experiences related to respectful care	Negative experiences related to communication and engagement in care	Negative experiences related to access to support			Negative experiences related to responsiveness
McMahon et al. (2014) [34]	Negative experiences related to respectful care					Negative experiences related to responsiveness
Dzomeku et al. (2017) [35]	Negative experiences related to respectful care	Negative experiences related to communication and engagement in care	Positive experiences related to access to support			Positive experiences related to responsiveness of staff
Oluoch-Aridi et al. (2018) [36]	Negative experiences related to respectful care		Negative experiences related to access to support			Negative experiences related to responsiveness
Kaye et al. (2015 [37])		Negative experiences related to communication and engagement in care		Women expectations regarding continuity of care Negative experiences related to continuity of care		
Madula et al. (2018) [38]	Negative experiences related to respectful care	Negative experiences related to communication and engagement in care				Positive experiences related to responsiveness of staff
Mselle et al. (2019) [39]	Positive and negative experiences related to respectful care	Significance of effective communication for satisfaction with services Negative experiences related to communication and engagement in care	Needs of women regarding birth companion and support from healthcare providers		Negative experiences related to pain management	Negative experiences related to responsiveness
Malachi et al. (2016) [40]						Negative experiences related to inadequate physical environment
Debela et al. (2021) [41]			Access to psychological support during labor			Negative experiences related to responsiveness
Burrowes et al. (2017) [42]	Negative experiences related to respectful care		Negative experiences related to access to support Preferences of women regarding birth companion	Negative experiences related to continuity of care		Negative experiences related to responsiveness
Bohren et al. (2017) [29]	Negative experiences related to respectful care Women perceived contributing factors of mistreatment		Positive experiences related to access to support			
Balde et al. (2017) [43]	Negative experiences related to respectful care					Negative experiences related to responsiveness
Jiru and Sendo (2021) [44]	Negative experiences related to respectful care		Positive experiences related to access to support			
Asrese (2020) [45]	Negative experiences related to respectful care	Negative experiences related to communication and engagement in care	Positive and negative experiences related to access to support			Negative experiences related to responsiveness
Lavender et al. (2021) [46]	Poor experiences were tolerated as long as their babies are fine Negative experiences related to respectful care					
Ojelade et al. (2017) [47]		Significance of effective communication for a positive childbirth experience	Needs of women related to access to support Negative experiences related to access to support			
Mgawadere et al. (2019) [48]	Negative experiences related to respectful care	Significance of effective communication for a positive childbirth experience	Infrastructural factors behind denial of birth companion Negative experiences related to access to support			Significance of feeling welcomed for positive experience
Orpin et al. (2018) [49]	Women perceived contributing factors of mistreatment					
Machira and Palamuleni (2018) [50]	Negative experiences related to respectful care		Negative experiences related to access to support			Negative experiences related to responsiveness
Gebremichael et al. (2018) [51]	Negative experiences related to respectful care		Positive experiences related to access to support	Negative experiences related to continuity of care	Negative experiences related to pain management	negative experiences related to Responsiveness

## Results

This qualitative meta-synthesis included 30 articles from nine African Sub-Saharan Low and Low-Middle Income Countries, including Ethiopia (*n* = 6), Ghana (*n* = 3), Guinea (*n* = 1), Kenya (*n* = 4), Malawi (*n* = 6), Nigeria (*n* = 3), Tanzania (*n* = 2), Uganda (*n* = 3), Tanzania and Zambia (*n* = 1), and Uganda and Nigeria (*n* = 1). The characteristics of the articles are summarised in Table 2. Most studies explored the experiences of women and their views concerning the quality of intrapartum care (*n* = 17). However, some studies included women’s partners as well (*n* = 3), different categories of healthcare providers, community health workers, and community leaders (*n* = 10). Based on the quality appraisal, the majority of included articles were of good quality. However, 3 out of 25 qualitative studies and 1 out of 5 mixed-methods studies had significant methodological limitations and lacked explanatory models. The summary of CASP and MMAT checklists is presented in supplementary files 3, 4 and 5.

**Table 2:** Characteristics of included articless

#	Reference	Country/ies	Method	Aim	Eligible study population
1	Afulani et al (2017) [21]	Kenya	Qualitative study Focus group discussions	To examine women’s facility-based childbirth experiences in a rural county in Kenya and aspects of care that contribute to a positive or negative birth experience.	58 women (postnatal)
2	Kumbani et al. (2012) [22]	Malawi	Qualitative study in-depth interviews	Women’s perceptions on perinatal care among the women delivered at a district hospital.	14 women (postnatal)
3	Jolly et al. (2019) [23]	Malawi	Qualitative study in-depth interviews and key-informant interviews	Women’s perceptions regarding respectful maternity care and the knowledge and understanding of the seven domains of the RMC Charter among healthcare providers	64 women (recruited from antenatal, intrapartum and postnatal clinics)
4	O’Donnell et al. (2014) [24]	Malawi	Qualitative study in depth interviews	Perceptions of women and HCPs of maternity care in a rural setting in Malawi.	33 women (postnatal)
5	Maya et al. (2018) [25]	Ghana	Exploratory qualitative study in depth interviews focus group discussions	Women’s perspectives of mistreatment during facility-based childbirth in the Ghanaian context	110 women
6	Dalinjong et al. (2018) [26]	Ghana	Qualitative part of a bigger convergent mixed-methods study Focus group discussions In-depth interviews	To assess the availability of basic inputs including drugs, supplies, equipment and emergency transport in health facilities and to explore women and health providers’ views on privacy and satisfaction with quality of care	women in postnatal period (number of women who participated in the FGD sessions is not provided)
7	Namujju et al. (2018) [27]	Uganda	Phenomenological qualitative study In-depth interviews and focus group discussions	To describe the childbirth experiences and the perceived meanings among postnatal mothers to broaden the information base for appropriate intervention development and individualized care during childbirth	25 women (postnatal)
8	Afulani et al. (2018) [28]	Kenya	Qualitative part of a bigger mixed methods study In-depth interviews and focus group discussions	Prevalence and determinants of birth companionship, and women and providers’ perceptions of it in health facilities in a rural County in Western Kenya	58 women (postnatal)
9	Bohren et al. (2017) [30]	Uganda and Nigeria	Exploratory qualitative study in depth interviews focus group discussions	To explore what “quality of care” means to childbearing women in Nigeria and Uganda,	Postnatal women (number of women who participated in the FGD sessions is not provided)
10	Kyaddondo et al. (2017) [32]	Uganda	Formative qualitative study in depth interviews focus group discussions	The experiences, expectations, and needs of urban Ugandan women in relation to good-quality facility childbirth.	85 women
11	Mehretie Adinew and Abera Assefa (2017) [33]	Ethiopia	Exploratory qualitative study In-depth interviews and focus group discussions	To explore why some women with previous experience of facility-based delivery care gave birth at home for their most recent child by in-depth understanding of women’s previous facility-based delivery experience, perspective towards health facilities and service providers with regard to delivery services	88 women
12	McMahon et al. (2014) [34]	Tanzania	Qualitative study (grounded theory) in depth interviews	To understand how rural Tanzanian women and their male partners describe disrespect and abuse experienced during childbirth in facilities and how they respond to abuse in the short or long-term	49 women
13	Dzomeku et al. (2017) [35]	Ghana	Exploratory qualitative research In-depth interviews	To explore women’s experiences with childbirth care in Kumasi, Ghana	56 women (attending antenatal or postnatal care)
14	Oluoch-Aridi et al. (2018) [36]	Kenya	Qualitative study in depth interviews and focus group discussions	The experiences and perceptions of women and healthcare workers regarding mistreatment during childbirth	46 women
15	Kaye et al. (2015) [37]	Uganda	Phenomenological qualitative study In-depth interviews	To gain a deeper understanding of mothers’ perspectives on quality of care (the structure, process and outcome of intrapartum care) particularly during duty handovers.	30 women (postnatal)
16	Madula et al. (2018) [38]	Malawi	Qualitative study in depth interviews	To examine the nature of communication in the maternity ward, and to identify facilitators and barriers to healthcare provider-patient communication	30 women (recruited from antenatal or postnatal clinic)
17	Mselle et al. (2019) [39]	Tanzania	Qualitative study in depth interviews and focus group discussions	The experiences of mothers and fathers in relation to mistreatment during childbirth in Tanzania.	13 women (postnatal)
18	Malachi et al. (2016) [40]	Kenya	Qualitative part of a bigger mixed methods study In-depth interviews and key informant interviews	To evaluate the institutional factors influencing women’s perception of quality intrapartum care.	women in postnatal period (number of women participated in the FGD session is not provided)
19	Debela et al. (2021) [41]	Ethiopia	Qualitative part of a bigger mixed methods study In-depth interviews	To explore underlying determinants of maternal satisfaction towards institutional delivery care among mothers who gave birth in public health facilities	36 women
20	Burrowes et al. (2017) [42]	Ethiopia	Qualitative study In-depth interviews	Women’s experiences of midwifery care during labor and to explore midwives’ understandings of patients’ rights and patient-centered care and their experiences with patient abuse and disrespect;	23 women
21	Bohren et al. (2017) [29]	Nigeria	Qualitative study In-depth interviews and focus group discussions	To explore women and providers’ experiences and perceptions of mistreatment during childbirth	75 women
22	Balde et al. (2017) [43]	Guinea	Qualitative study In-depth interviews and focus group discussions	To explore the perceptions and experiences of women and HCPs of mistreatment during childbirth	109 women
23	Jiru and Sendo (2021) [44]	Ethiopia	Exploratory qualitative study In-depth interviews	To explore women’ and midwives’ perceptions of compassionate and respectful care during facility-based delivery	12 women (postnatal)
24	Asrese (2020) [45]	Ethiopia	Qualitative part of a bigger mixed methods study In-depth interviews	To assess the quality of intrapartum care experienced by mothers at health centers	25 women (postnatal)
25	Lavender et al. (2021) [46]	Tanzania and Zambia	Qualitative study (grounded theory) In-depth interviews	Exploring care through multiple lenses enabling a more comprehensive understanding of relational contributors to experiences through examining the intrapartum experiences of women, partners, different health-providers and key stakeholders	48 women (postnatal)
26	Ojelade et al. (2017) [47]	Nigeria	Qualitative study in depth interviews and focus group discussions	Women’s needs for communication and emotional support during facility- based childbirth	77 women
27	Mgawadere et al. (2019) [48]	Malawi	qualitative study Focus group discussions and key-informant interviews	To explore women’s and healthcare provider’s perspectives of what quality of care during childbirth means to them	134 women (postpartum)
28	Orpin et al. (2018) [49]	Nigeria	Phenomenological qualitative study Focus group discussions	To explore the women experiences of disrespect and abuse during pregnancy, childbirth, and in the postnatal period and its impact on the future use of health facilities for maternity care.	32 women (postnatal)
29	Machira and Palamuleni (2018) [50]	Malawi	Qualitative study Focus group discussions	Women’s perspectives on the quality of maternal health care services in Malawi	58 women
30	Gebremichael et al. (2018) [51]	Ethiopia	Phenomenological qualitative study Focus group discussions	Women’s experience of disrespect and abuse during childbirth at health facilities	62 women

We present the women’s experiences and expectations regarding quality intrapartum care categorised according to the themes adopted from the WHO framework for a positive childbirth experience; dignified care and respect, communication and meaningful engagement in care, access to emotional support during childbirth, continuity of care, pain management, and responsiveness of health facility setting and health services. Our findings indicate that women in Sub-Saharan LLMICs need clinical and non-clinical staff to treat them with respect and in a non-discriminatory and non-abusive manner. In addition, women wanted to feel welcomed throughout their stay in health facilities. Women described the need to be meaningfully involved in their care and to have open and effective communication with their care providers which helped them prepare for labour. Women also wanted to be emotionally and physically supported by care providers and their birth companions. The findings show that women expect to receive timely care and be monitored closely in a safe environment at health facilities.

### Dignified care and respect

Women demanded to be treated with respect during childbirth and expected healthcare providers to be non-judgmental [23], kind [22], and respectful [21]. For a positive childbirth experience, women described needing respectful and dignified intrapartum care while maintaining their privacy and wanting to be meaningfully engaged in their care [32]. In addition to health care providers (HCPs), the positive attitude of non-clinical staff, such as cleaners and security guards also contributed to a positive experience [21]. Women described dignified care that involved physical support [21].

*When I was in labor, a nurse brought me porridge for me to have energy during delivery of the baby. I saw that I was respected* [22]

Disrespectful care was also reported by women in LLMICs who were verbally and physically abused during childbirth and suffered discrimination as a result of their age, ethnicity, literacy level, and socioeconomic status [21, 25, 27, 29, 35, 36, 42–44, 48–51].

*Respectful. The first thing which comes to my mind ... the client must be respected. Respected that is ... to receive care ... not (taking into account) age, worth, colour or religion* [23].

Verbal and physical abuse included shouting, yelling, ridicule, judgmental remarks from healthcare providers, and slapping and whipping of women during childbirth. In few studies, however, women considered the abusive behaviour of healthcare providers as a normative behaviour [49] or they expected to be shouted at [22, 25] or provided sociocultural and contextual justifications for their behaviour such as encouragement of women, stress, and poor working conditions [25, 29, 30, 48]. Women also encountered discrimination as well based on their ethnicity/tribes, age, literacy level, and socioeconomic status [21, 25, 34, 36, 38, 46]. A study conducted in Zambia and Tanzania classified discrimination into two categories; direct and indirect, direct discrimination includes discriminatory incidents that happen to women during childbirth whereas indirect discrimination was defined as when women received poor quality of care caused by policies that are meant for everyone such as the assignment of professionals to central and referral facilities instead of rural areas [46]. Studies showed that young girls giving birth or unmarried women received judgmental remarks from HCPs [25, 36]. Women demanded to be treated impartially without discrimination and considered being treated by non-judgmental HCPs as essential for a positive childbirth experience [23].

Women in LLMICs also reported experiencing a lack of privacy during childbirth and unnecessary physical exposure due to contextual factors such as crowded wards and poor infrastructure at health facilities [21, 23, 30, 32, 33, 45, 46].

*Due to the lack of infrastructure and congestion of hospitals, it was hard to maintain the privacy of women in such context so it was not uncommon to have several women giving birth in the same room - so women need privacy during childbirth with curtains or cubicles* [32]

However, one study indicated that privacy was not a big concern for women in that context compared to not having care [23]. In addition, women could not complain about the lack of privacy because they feared retribution from care providers [46].

### Communication and meaningful engagement in care

In half of the included studies, women described their experiences and expectations regarding communication with health providers and engagement in care. Women in LLMICs reported that open, effective, clear, two-way communication where HCPs used positive language and were able to ask questions as an important aspect of quality care and satisfaction with delivery services [21, 23, 30, 32, 39, 47, 48]. A women stated “*Communication is very, very important…it is everything”* [47]. They appreciated it when HCPs sought their consent and gave them regular updates about their progress in labour [21, 23] and they referred to having open communication as ‘friendship’ with care providers [30].

*They [healthcare providers] should continuously ask questions... ‘how do you feel,’ ‘how are you feeling now.’ It’s not supposed to just be the woman that will be telling them ‘please come check on me’...they [healthcare providers] should be continuously telling the woman ‘this is your condition,’ and educate them *[47].

Women cited poor communication during childbirth [22, 24, 27, 32, 39, 43] that included the inability to ask questions [38, 45], HCPs not introducing themselves or not using women’s names [21], communication difficulties for women living with disability [38], and communication in languages other than their mother tongue [38]. Women also spoke of a lack of information that made them feel unprepared and caused stress during the labour [30]. In addition, communication gaps resulted in misinterpretation of providers’ motivations [32].

Studies indicated that women were rarely involved in decision-making in LLMICs [24, 27, 35, 39, 42, 50]. For instance, women did not understand why care providers opted to use a specific management plan [24, 27, 35]. In addition, women were not involved in choosing their delivery positions [39].

*“The midwives did not even engage me in any discussions over my childbirth process. They never told or asked me about anything”* [35].

Women in LLMICs wanted to be listened to and be meaningfully involved in decision-making in actions affecting their care such as preferred labour position, treatment options, and others [32, 39].

### Access to emotional support during childbirth

The experiences and expectations of women regarding emotional support through labour were detailed in the majority of studies. According to the voices of women in the papers included in this review, labour constituted an important and stressful occasion that substantiates emotional support throughout their stay at the health facilities. Women wanted to be cared for and encouraged to go through labour with a birth companion of their choice [21, 26–28, 30, 32, 47].

*Good quality of care is when you come to a health facility, you are received, they know what that moment means to you and that of your child and the kind of reception they give you as a mother that wants to deliver her child. They receive you warmly, encourage you. .. what matters most is that when you come into a health facility, there’s this confidence derived that assures one that she’s in good hands* [30].

Women in LLMICs were accompanied by their mothers, sisters, mothers-in-law, husbands, and *doula* (a traditional birth attendant or an older woman from their communities) [27, 28, 32]. Husbands in the majority of facilities where studies took place were not allowed inside the labour room [30, 32, 47]. This was mainly due to the need to maintain the privacy of other women because of shared labour rooms [28, 48].

Some women did not want their husbands and partners to be present during labour, [21, 27, 48] others preferred female companions [28], while others wished their husbands to be present to provide emotional support during labour [30, 39, 47].

*I think women should be allowed their husbands in...my husband was right there with me; my first delivery, it was painful but with his encouragement, he was there holding my hands, doing this, even when the doctors were telling madam push, push, I didn’t listen to the doctors but when my husband say madam push, push, that is when I started pushing. I think it is a psychological thing when your husband is right there with you* [30].

In the majority of the studies, women mentioned the need to have a birth companion to support the mother and baby’s basic needs, such as the provision of food and drinks, going to the toilet, initiating breastfeeding, cleaning the baby, and assistance with mobility, [21, 27, 28, 30, 32]. Fewer studies quoted the need for emotional support from birth companions [32, 39]. Instead, women expected healthcare providers, especially midwives to offer emotional support during labour (one-to-one care) since in most contexts birth companions were not allowed inside the labour room [30, 32, 42, 45, 47].

*I came here and met three midwives on duty. They actually supported me. They stood by me until the baby was delivered. One of them even held my hands during delivery and encouraged me throughout the process. They remained with me and responded well to all my numerous requests and questions. They never neglected me and I really appreciated them for that* [35].

Women in LLMICs cited a lack of supportive healthcare providers during labour [22, 25, 26, 29, 33, 36, 39, 41, 44, 45, 48, 51]. According to women, the factors that affect the responsiveness of HCPs included ignorance, being busy with irrelevant matters such as phone calls, heavy workloads, inadequate staff numbers, and poor working conditions [25, 30, 36, 39, 44, 46].

*(.. .) So, if someone feels that the baby is coming and it’s time for delivery, she may call for help but only to be disappointed by nurses who think that she is pretending. But because they are busy with their own things, they don’t pay attention (.. .)* [39]

### Continuity of care

Only five studies included elements related to women’s experiences of continuity of care during childbirth [30, 32, 37, 42, 51]. There was variability in women’s preferences of models of continuity of care. One study described women’s preferences for a single provider throughout their pregnancy journey to build trust and reliability of information [30]. Other women indicated their desire for a team of providers with diverse skills to manage their childbirth and respond to complications [32, 37].

Despite the importance of continuity of services throughout their stay at the health facility, women experienced an interruption of care between shifts, lower quality care, and a lack of monitoring during night shifts [37, 42, 51].

*“It was not done well. It was usually very brief. Often the doctors did not even look at you, let alone examine you. .. Yet the doctors change all the time. They do not seem to be working as one healthcare team.”* [51]

In addition, women observed a lack of handover between shifts which resulted in women feeling uninvolved or abandoned and led to poor communication of critical information between providers causing delays.

*Some doctors make wrong diagnoses or make wrong decisions. And when one group comes to replace the one that has been treating you, they change the treatment, without asking you any questions or examining you. One team tells that you are for an operation, and another team cancels the operation or tells you that nothing was written. Nobody asks for your opinion and rarely do they answer your questions during rounds* [37].

### Pain management

Pain relief is crucial for a positive experience and satisfaction with services. A few studies included data concerning women’s experiences of pain management in labour [21, 22, 27, 39, 51]. A study conducted in Uganda indicated that women perceived labour pain as natural and inevitable, therefore, they did not expect to have medication to manage such pain [27]. However, they expected to get advice on how to deal with pain [22].

*I think no need of medicine, because it is natural. I think even if they give you some medicine for pain, contractions would still come because the baby has to come out. I think the drugs cannot reduce those pains...every other woman goes through that* [27]

Women described care providers as uncaring and lacking sympathy when they did not provide pain management advice [21, 22, 39, 51]. Women reported enduring surgical interventions without local anaesthesia [32, 39].

*( ... ) if they had responded in time, maybe my parts wouldn’t have been torn. Despite the fact that I was torn, they still stitched me without any pain killer and when I tried to refuse, I was told that I did not bring the required drugs and that if I did not want to be stitched without pain killer I should pay money for the drug and wait for them to go and buy the drugs. So to be honest, I will never ever return to that hospital again* [39]

### Responsiveness of health facility setting and health services

Women perceived timely assessment and management as quality care [23, 24, 27, 30, 32]. A woman described quality care as follows *“[Good quality care is] when you have been received well by the staff at the hospital, and they have helped you quickly”*[24]. They emphasised the importance of feeling welcomed by healthcare providers as soon as they arrived at the hospital and throughout their stay for a positive childbirth experience [21, 22, 24, 25, 32, 48]. Women also valued delivery services available around the clock whenever they needed them and that someone was there to open hospital gates 24/7 [21, 23, 24]. Women appreciated it when healthcare providers hastened to examine them when they arrived and provided the needed care [21, 23, 24, 35, 38, 51]. In addition, they appreciated it when care providers went out of their way to help them such as midwives providing their personal time and drugs to support women [21].

*They treated me with respect because they took good care of me until I delivered and did everything well. After delivery they gave me water for bathing, later I was taken to the bed and they gave me the baby to breastfeed* [21]

Women in LLMICs, however, experienced a lack of timely assessment and delays while using health services [21, 22, 27, 33, 39, 41, 44, 45, 48, 51]. In addition, women gave birth unattended by HCPs at health facilities due to unavailable, busy or unresponsive staff [22, 25, 36, 43, 51].

*I was examined and told my labour is at an early stage ... at that point, my baby was on the way out but I was restricted to stay in my left side ...I told my care provider I am urged to push down and requested for help ...he said I just examined you (you are not yet ready) and ignored me and continued playing with his mobile phone ...the urge to push down was irresistible, I then turned on my back by myself and gave birth (.. .) *[51]

Women highlighted the benefit of having a conducive physical environment and the availability of needed supplies for a positive childbirth experience [21]. Women emphasised the need to have clean facilities (especially delivery wards and bathrooms) with sunlight, access to water, electricity, and sanitation services, adequate beds, uncrowded wards, adequate space and curtains for privacy, and access to bed nets [21, 30, 32]. There were also descriptions of negative experiences related to the inadequate physical environment including crowded rooms [29, 30, 40, 45] insufficient beds [29, 30, 36, 39, 50], lack of access to water and food [21, 50], dark labour rooms with no natural sunlight [21, 30] and unclean premises [30, 43].

*[A]fter delivery there is a room we were taken to sleep, there was no light, no windows, no beddings and we were to stay there feeling cold till morning. That is the worst I experienced... [The room had windows with no glass in them], and it was very cold and we were about three mothers with newly born babies. Cats were just entering through that window and just walking in that hospital...there was lack of security *[21].

## Discussion

Our meta-synthesis showed that woman-centred care, incorporating respect and meaningful engagement is necessary for a positive intrapartum experience for women in LLMICs. Woman-centred maternity care encompasses effective communication, respect and dignity, and emotional support [10]. These dimensions shape the care experiences of women, how they perceive quality care and their satisfaction with services [11]. Our findings show that women in LLMICs desire the same intrapartum and immediate postpartum care as women in other countries during this period [14]. However, priorities regarding the components of quality care for women and the urgency to intervene differed in this context given the socio-cultural norms and available resources. For instance, despite the growing interest in the promotion of respectful care [52], women still encounter disrespectful care in health facilities in LLMICs including physical and verbal abuse, discrimination, and lack of privacy. In line with previous studies, our review indicates that adolescent mothers and unmarried women were more susceptible to mistreatment [53]. In addition, studies indicated poor communication between women and healthcare providers, non-consensual care, and women were rarely involved in their care. A systematic review suggested that women in low-income countries are less likely to expect involvement in care and to demand their rights in the decision-making [54]. Playing a passive role in childbirth could be attributed to cultural and gender norms and the low empowerment of women in the LLMICs [4]. Long-term interventions are required to empower women in these settings, provide them with knowledge of their right to participate in decision-making, and give them the self-assurance to assert those rights [55]. A randomised controlled trial was conducted in Tanzania and Malawi to evaluate the effect of group antenatal care (ANC) versus individual ANC i.e. standard care on the empowerment of women measured by the Pregnancy-Related Empowerment Scale (PRES) [56]. This scale measures how effectively pregnant women engage in decision-making, communicate with and feel connected to their peers and healthcare professionals [56]. The group ANC included a two-hours interactive group session of education and support for pregnant women in addition to a private consultation with the midwife to monitor the pregnancy [56]. The study showed that pregnant women who participated in the group ANC had higher PRES scores in some contexts, especially in rural and poor settings where health facilities provide low-quality maternity services [56]. More research is needed to investigate the feasibility of implementing a similar model of care in other countries in LLMICs.

Our findings indicate that women in LLMICs were denied a companion of their choice. In the context of LLMICs, a birth companion offers the woman emotional and practical support and serves as an advocate, expressing her preferences to healthcare professionals and defending her choices. A systematic review indicated that women who had continuous one-to-one support during childbirth had better outcomes than those who lacked support during spontaneous vaginal delivery [31]. Supported women have less need for analgesia, had shorter labours and were satisfied with the intrapartum services they received [31]. The same systematic review suggested that having continuous support throughout labour may promote respectful care and safeguard against the mistreatment of women during childbirth [31].

As illustrated in our findings, the non-clinical aspects of care play an essential role in shaping the experiences of care, satisfaction with services, and future care-seeking behaviours. According to a systematic review, even when evidence-based clinical criteria are followed, maternity services are deemed low quality if they are disrespectful to the women receiving them [57]. Nevertheless, investment in interventions to improve non-clinical aspects of care such as respectful care, meaningful involvement of women in their care, and effective communication during childbirth are often not a priority in LLMICs settings [13]. Despite the recent global recognition of the significance of respectful care, a lack of political will and quality maternity care guidelines, in addition to limited resources in LLMICs have put interventions to enhance women’s experiences at the bottom of the agenda [4, 57, 58].

Our findings indicate that most women gave birth in health facilities with limited infrastructure and resources indicating the need for government investment in LLMICs. Nevertheless, there are cost-effective interventions that can help improve the quality of care such as training healthcare providers on interpersonal communication, mentoring, and setting accountability systems where women can voice their experiences and expectations [58]. Women reported fewer occurrences of disrespectful care, according to a systematic review of studies from Kenya, Tanzania, Sudan, and South Africa that examined the impact of implementing measures to improve respectful maternity care [58]. A before-and-after intervention study evaluated the impact of implementing a bundle of respectful maternity care policies in 13 facilities in Kenya including training of care providers, capacity building of quality improvement teams at facilities, caring for Carers which included counselling of care providers on coping with stress, and community activities including community workshops to educate the public about their rights [59]. This study revealed a decrease in the incidence of observed disrespectful care and abuse of women [59]. These interventions were effective as the package targeted health facilities, women, care providers, and the community which acknowledges the interconnectivity between these different actors and the socio-cultural environment at local facilities and community [58, 59]. For instance, the same study showed a discrepancy between reported and observed disrespectful care due to the low expectations of women regarding their care. Therefore, it is essential to have interventions to raise women’s awareness regarding their rights [59].

There are different models regarding the organisation of care during pregnancy, including midwife-led continuity of care, obstetrician-provided care, family doctor-provided care, or shared model of care where health services are provided by a team of providers [60]. Our findings indicate the paucity of evidence with regard to the continuity of care during childbirth in LLMICs. A systematic review showed that women who had midwifery-led care had an increased likelihood of a spontaneous vaginal delivery and reduction in pre-term labour; however, the evidence was lacking the long-term maternal and baby wellbeing outcomes [61]. Our review indicated that women preferred having a single care provider throughout their pregnancy journey to build their trust, confidence, and smooth transition to parenthood. A study that assessed the quality of services provided by midwives in Uganda indicated that midwives provided low-quality services for women [62]. According to the study, weak knowledge and skills of midwives are attributed to inadequate in-service training, lack of supportive supervision, and absence of written guidelines [62]. A systematic review examining the reasons why midwives do not provide quality services in Low- and Middle-Income Countries showed that weak or absent midwifery regulations and heavy workloads were major barriers [63]. Besides, short training courses that midwives receive before their midwifery practice as a temporary solution to improve coverage with skilled birth attendance have a negative impact on the quality of care they provide, especially for those working in remote areas without support from the health system [63]. These limitations related to midwifery education and regulations, the skills of midwives in LLMICs, and the supportive environment can probably jeopardise the application of midwife-led continuity of care in LLMICs. Further research is required about the feasibility and effectiveness of implementing a midwife-led approach in the context of health facilities in LLMICs, short and long-term outcomes given the current limitations.

Our review did not specifically focus on the experiences and expectations of women in fragile settings. However, a third of the included articles were conducted in countries classified as fragile states [64] including Ethiopia, Guinea, and Nigeria. Health systems in fragile states suffer unique challenges, including insecurity, reliance on international support, weak leadership and management, and insufficient human and financial resources for health [65, 66]. In these contexts, midwives play an important role in providing maternal care given their knowledge, skills, and closeness to communities [67]. Evidence shows that investments in improving the quality of midwifery education and regulations are cost-efficient and can enhance the quality of maternity care and woman-centred outcomes in humanitarian settings and stable developing settings [67, 68]. Our review indicates that there is a paucity of research that explores the views of women in fragile states regarding quality maternity care, the status, and the contextual factors that affect woman-centred outcomes. Prioritising context-relevant interventions based on the needs and expectations of women in general and especially marginalised women resonates with a key cornerstone of the Sustainable Development Goals, reducing inequities in access to quality services, and leaving no one behind [69].

### Limitations

Our qualitative meta-synthesis has a few limitations. Few articles had minimal methodological rigor, in addition, the findings were rather descriptive and lacked explanatory models. Furthermore, the authors of the included articles were not explicit about theoretical frameworks and forms of inquiry. We included all eligible articles, even if they were of low quality, as an attempt to incorporate all women’s voices from different contexts. However, the reported themes may have been limited by the quality of the original articles. We also noticed that the majority of included articles lacked researcher reflexivity and they did not fully describe study limitations. A lack of detail concerning the research methodology can result in questions regarding the trustworthiness of the findings and the possible misinterpretation of participants’ voices.

## Implications for policy and practice

Quality health systems should cater to the needs of the population they serve. The planning of interventions to improve the quality of maternity care must be based on communication with women and women groups to identify context-specific factors to optimise implementation and outcomes. Women must be meaningfully engaged in the design of health services, accountability frameworks, and evaluation of maternal services. Designing systems that capture women’s needs can be challenging in the context of LLMICs unless there is a commitment from policymakers, health programs, and practitioners.

Our findings indicate that women in LLMICs received sub-quality intrapartum care and global standards for woman-centred care were often compromised. Given the limitations that health systems in LLMICs have, including lack of quality maternal health guidelines, limited health financing, and resources, we suggest that the global standards for a positive childbirth experience are hard to achieve in these settings. Therefore, a set of minimum indicators for woman-centred outcomes that work in the context of LLMICs is needed. Given the weaknesses of health information systems in LLMICs, we suggest that a minimum set of indicators be incorporated in the WHO standards for quality maternal and newborn care in health facilities [70] as core indicators designed for these settings. Furthermore, we recommend actionable strategies to enhance the quality of maternity care based on women’s needs and preferences. Indicators can quantitatively measure women’s care experiences including dignified and respectful care, autonomy, effective communication, involvement in care, access to emotional support, and supportive care and physical environment. We recommend that the indicators related to women’s care experiences be integrated with national health indicators in LLMICs to provide a database that can be used to monitor countries’ progress in improving the quality of maternity health services. We are cognizant of the limitations of national health information systems in LLMICs. The inclusion of simple feedback mechanisms to report women’s satisfaction with health services using phone text messages could be useful. In addition, countries can explore the possibility of implementing results-based financing to healthcare providers to improve the quality of health information, including indicators to monitor care experiences [71, 72].

## Conclusion

To improve care seeking and satisfaction with health services, woman-centred care, where women and their newborns are at the centre of their care is necessary for a positive childbirth experience. Women must be meaningfully engaged in the design of health services, accountability frameworks, and evaluation of maternal services. Further research is needed to set minimum indicators for woman-centred outcomes that work in the context of sub-Saharan African LLMICs along with actionable strategies to enhance the quality of maternity care based on women’s needs and preferences.

## Supplementary Information

**Additional file 1: Supplementary file 1.** Medline database search strategy.

**Additional file 2: Supplementary file 2.** Inclusion and exclusion criteria.

**Additional file 3: Supplementary file 3.** Critical appraisal of included articles – CASP checklist.

**Additional file 4: Supplementary file 4.** Critical appraisal of included articles – CASP checklist.

**Additional file 5: Supplementary file 5.** Critical appraisal of included articles – MMAT checklist.

## Data Availability

The datasets used and/or analysed during the current study available from the corresponding author on reasonable request.

## Abbreviations

ANC: Antenatal care
CASP: Critical Appraisal Skills Programme
HCPs: Health care providers
LLMICs: Low and Low Middle-Income Countries
MMAT: Mixed Methods Appraisal Tool
PRES: Pregnancy-Related Empowerment Scale
PRISMA: Preferred Reporting Items for Systematic Reviews and Meta-Analyses
PROSPERO: The International Prospective Register of Systematic Reviews
SBA: Skilled birth attendant
SDGs: Sustainable Development Goals
SSA: Sub-Saharan Africa
WHO: World Health Organisation

## References

[1] World Health Organisation (2017). Global health observatory data: maternal mortality.

[2] Mselle LT, Moland KM, Mvungi A, Evjen-Olsen B, Kohi TW (2013). Why give birth in health facility? Users’ and providers’ accounts of poor quality of birth care in Tanzania. BMC Health Serv Res.

[3] Graham WJ, McCaw-Binns A, Munjanja S (2013). Translating coverage gains into health gains for all women and children: the quality care opportunity. PLoS Med.

[4] Bohren MA, Tunçalp Ö, Miller S (2020). Transforming intrapartum care: respectful maternity care. Best Pract Res Clin Obstet Gynecol.

[5] Adegoke AA, Hofman JJ, Kongnyuy EJ, Van Den Broek N (2011). Monitoring and evaluation of skilled birth attendance: a proposed new framework. Midwifery.

[6] Harvey SA, Blandón YCW, McCaw-Binns A, Sandino I, Urbina L, Rodríguez C (2007). Are skilled birth attendants really skilled? A measurement method, some disturbing results and a potential way forward. Bull World Health Organ.

[7] World Health Organization (2017). Global health observatory data: skilled attendants at birth.

[8] Mathai M (2011). To ensure maternal mortality is reduced, quality of care needs to be monitored and improved alongside increasing skilled delivery coverage rates. BJOG.

[9] Munabi-Babigumira S, Glenton C, Lewin S, Fretheim A, Nabudere H (2017). Factors that influence the provision of intrapartum and postnatal care by skilled birth attendants in low-and middle-income countries: a qualitative evidence synthesis. Cochrane Database Syst Rev.

[10] Rishard M, Fahmy FF, Senanayake H, Ranaweera AKP, Armocida B, Mariani I (2021). Correlation among experience of person-centered maternity care, provision of care and women’s satisfaction: cross sectional study in Colombo, Sri Lanka. PLoS One.

[11] Afulani PA, Diamond-Smith N, Golub G, Sudhinaraset M (2017). Development of a tool to measure person-centered maternity care in developing settings: validation in a rural and urban kenyan population. Reprod Health.

[12] Oladapo O, Tunçalp Ö, Bonet M, Lawrie T, Portela A, Downe S (2018). WHO model of intrapartum care for a positive childbirth experience: transforming care of women and babies for improved health and wellbeing. BJOG.

[13] World Health Organization. WHO recommendations on intrapartum care for a positive childbirth experience. World Health Organization; Geneva. 2018. Report No.: 924155021X. 30070803

[14] Downe S, Finlayson K, Oladapo O, Bonet M, Gülmezoglu AM (2018). What matters to women during childbirth: a systematic qualitative review. PLoS One.

[15] Dawson AJ (2019). Meta-Synthesis of qualitative research. Journal: Handbook of Research Methods in Health Social Sciences.

[16] Moher D, Liberati A, Tetzlaff J, Altman DG, Group* P (2009). Preferred reporting items for systematic reviews and meta-analyses: the PRISMA statement. Ann Intern Med.

[17] Programme CAS (2014). CASP checklists. Critical Appraisal Skills Programme (CASP): making sense of evidence.

[18] Hong Q, Pluye P, Fàbregues S, Bartlett G, Boardman F, Cargo M (2018). Mixed methods appraisal tool (MMAT) version 2018: user guide.

[19] Carroll C, Booth A, Cooper K (2011). A worked example of” best fit” framework synthesis: a systematic review of views concerning the taking of some potential chemopreventive agents. BMC Med Res Methodol.

[20] Carroll C, Booth A, Leaviss J, Rick J (2013). “Best fit” framework synthesis: refining the method. BMC Med Res Methodol.

[21] Afulani PA, Kirumbi L, Lyndon A (2017). What makes or mars the facility-based childbirth experience: thematic analysis of women’s childbirth experiences in western Kenya. Reprod Health.

[22] Kumbani LC, Chirwa E, Odland J, Bjune G (2012). Do malawian women critically assess the quality of care? A qualitative study on women’s perceptions of perinatal care at a district hospital in Malawi. Reprod Health.

[23] Jolly Y, Aminu M, Mgawadere F, van den Broek N (2019). “We are the ones who should make the decision”–knowledge and understanding of the rights-based approach to maternity care among women and healthcare providers. BMC Pregnancy Childbirth.

[24] O’Donnell E, Utz B, Khonje D, Van Den Broek N (2014). ‘At the right time, in the right way, with the right resources’: perceptions of the quality of care provided during childbirth in Malawi. BMC Pregnancy Childbirth.

[25] Maya ET, Adu-Bonsaffoh K, Dako-Gyeke P, Badzi C, Vogel JP, Bohren MA (2018). Women’s perspectives of mistreatment during childbirth at health facilities in Ghana: findings from a qualitative study. Reprod Health Matters.

[26] Dalinjong PA, Wang AY, Homer CS (2018). Are health facilities well equipped to provide basic quality childbirth services under the free maternal health policy? Findings from rural northern Ghana. BMC Health Serv Res.

[27] Namujju J, Muhindo R, Mselle LT, Waiswa P, Nankumbi J, Muwanguzi P (2018). Childbirth experiences and their derived meaning: a qualitative study among postnatal mothers in Mbale regional referral hospital, Uganda. Reprod Health.

[28] Afulani P, Kusi C, Kirumbi L, Walker D (2018). Companionship during facility-based childbirth: results from a mixed-methods study with recently delivered women and providers in Kenya. BMC Pregnancy Childbirth.

[29] Bohren MA, Vogel JP, Tunçalp Ö, Fawole B, Titiloye MA, Olutayo AO (2017). Mistreatment of women during childbirth in Abuja, Nigeria: a qualitative study on perceptions and experiences of women and healthcare providers. Reprod Health.

[30] Bohren MA, Titiloye MA, Kyaddondo D, Hunter EC, Oladapo OT, Tunçalp Ö (2017). Defining quality of care during childbirth from the perspectives of nigerian and ugandan women: a qualitative study. Int J Gynecol Obstet.

[31] Bohren MA, Hofmeyr GJ, Sakala C, Fukuzawa RK, Cuthbert A (2017). Continuous support for women during childbirth. Cochrane Database Syst Rev.

[32] Kyaddondo D, Mugerwa K, Byamugisha J, Oladapo OT, Bohren MA (2017). Expectations and needs of ugandan women for improved quality of childbirth care in health facilities: a qualitative study. Int J Gynecol Obstet.

[33] Mehretie Adinew Y, Abera Assefa N (2017). Experience of facility based childbirth in rural Ethiopia: an exploratory study of women’s perspective. J Pregnancy.

[34] McMahon SA, George AS, Chebet JJ, Mosha IH, Mpembeni RN, Winch PJ (2014). Experiences of and responses to disrespectful maternity care and abuse during childbirth; a qualitative study with women and men in Morogoro Region, Tanzania. BMC Pregnancy Childbirth.

[35] Dzomeku VM, van Wyk B, Lori JR (2017). Experiences of women receiving childbirth care from public health facilities in Kumasi. Ghana Midwifery.

[36] Oluoch-Aridi J, Smith-Oka V, Milan E, Dowd R (2018). Exploring mistreatment of women during childbirth in a peri-urban setting in Kenya: experiences and perceptions of women and healthcare providers. Reprod Health.

[37] Kaye DK, Nakimuli A, Kakaire O, Osinde MO, Mbalinda SN, Kakande N (2015). Gaps in continuity of care: patients’ perceptions of the quality of care during labor ward handover in Mulago hospital, Uganda. BMC Health Serv Res.

[38] Madula P, Kalembo FW, Yu H, Kaminga AC (2018). Healthcare provider-patient communication: a qualitative study of women’s perceptions during childbirth. Reprod Health.

[39] Mselle LT, Kohi TW, Dol J (2019). Humanizing birth in Tanzania: a qualitative study on the (mis) treatment of women during childbirth from the perspective of mothers and fathers. BMC Pregnancy Childbirth.

[40] Malachi Z, Omuga B, Mirie W (2016). Institutional factors influencing women’s perception of quality of intrapartum care in Naivasha County Hospital labour ward, Kenya. Afr J Midwifery Women’s Health.

[41] Debela AB, Mekuria M, Kolola T, Bala ET, Deriba BS (2021). Maternal satisfaction and factors associated with institutional delivery care in central Ethiopia: a mixed study. Patient Prefer Adherence.

[42] Burrowes S, Holcombe SJ, Jara D, Carter D, Smith K (2017). Midwives’ and patients’ perspectives on disrespect and abuse during labor and delivery care in Ethiopia: a qualitative study. BMC Pregnancy Childbirth.

[43] Balde MD, Bangoura A, Sall O, Soumah AM, Vogel JP, Bohren MA (2017). Perceptions and experiences of the mistreatment of women during childbirth in health facilities in Guinea: a qualitative study with women and service providers. Reprod Health.

[44] Jiru HD, Sendo EG (2021). Promoting compassionate and respectful maternity care during facility-based delivery in Ethiopia: perspectives of clients and midwives. BMJ open.

[45] Asrese K (2020). Quality of intrapartum care at health centers in Jabi Tehinan district, North West Ethiopia: clients’ perspective. BMC Health Serv Res.

[46] Lavender T, Bedwell C, Kasengele CT, Kimaro D, Kuzenza F, Lyangenda K (2021). Respectful care an added extra: a grounded theory study exploring intrapartum experiences in Zambia and Tanzania. BMJ Global Health.

[47] Ojelade OA, Titiloye MA, Bohren MA, Olutayo AO, Olalere AA, Akintan A (2017). The communication and emotional support needs to improve women’s experience of childbirth care in health facilities in Southwest Nigeria: a qualitative study. Int J Gynecol Obstet.

[48] Mgawadere F, Smith H, Asfaw A, Lambert J, van den Broek N (2019). “There is no time for knowing each other”: quality of care during childbirth in a low resource setting. Midwifery.

[49] Orpin J, Puthussery S, Davidson R, Burden B (2018). Women’s experiences of disrespect and abuse in maternity care facilities in Benue State, Nigeria. BMC Pregnancy Childbirth.

[50] Machira K, Palamuleni M (2018). Women’s perspectives on quality of maternal health care services in Malawi. Int J Women’s Health.

[51] Gebremichael MW, Worku A, Medhanyie AA, Edin K, Berhane Y (2018). Women suffer more from disrespectful and abusive care than from the labour pain itself: a qualitative study from women’s perspective. BMC Pregnancy Childbirth.

[52] Vogel JP, Bohren MA, Tunçalp Ó¦, Oladapo OT, Gülmezoglu A (2016). Promoting respect and preventing mistreatment during childbirth. BJOG.

[53] Bohren MA, Vogel JP, Hunter EC, Lutsiv O, Makh SK, Souza JP (2015). The mistreatment of women during childbirth in health facilities globally: a mixed-methods systematic review. PLoS Med.

[54] Shakibazadeh E, Namadian M, Bohren MA, Vogel JP, Rashidian A, Nogueira Pileggi V (2018). Respectful care during childbirth in health facilities globally: a qualitative evidence synthesis. BJOG.

[55] Elmusharaf K, Byrne E, O’Donovan D (2015). Strategies to increase demand for maternal health services in resource-limited settings: challenges to be addressed. BMC Public Health.

[56] Patil CL, Klima CS, Leshabari SC, Steffen AD, Pauls H, McGown M (2017). Randomized controlled pilot of a group antenatal care model and the sociodemographic factors associated with pregnancy-related empowerment in sub-saharan Africa. BMC Pregnancy Childbirth.

[57] Miller S, Abalos E, Chamillard M, Ciapponi A, Colaci D, Comandé D (2016). Beyond too little, too late and too much, too soon: a pathway towards evidence-based, respectful maternity care worldwide. Lancet.

[58] Downe S, Lawrie TA, Finlayson K, Oladapo OT (2018). Effectiveness of respectful care policies for women using routine intrapartum services: a systematic review. Reprod Health.

[59] Abuya T, Ndwiga C, Ritter J, Kanya L, Bellows B, Binkin N (2015). The effect of a multi-component intervention on disrespect and abuse during childbirth in Kenya. BMC Pregnancy Childbirth.

[60] Sandall J, Devane D, Soltani H, Hatem M, Gates S (2010). Improving quality and safety in maternity care: the contribution of midwife-led care. J Midwifery Women’s Health.

[61] Sandall J, Soltani H, Gates S, Shennan A, Devane D (2016). Midwife-led continuity models versus other models of care for childbearing women. Cochrane Database Syst Rev.

[62] Kaye D (2000). Quality of midwifery care in Soroti district, Uganda. East Afr Med J.

[63] Filby A, McConville F, Portela A (2016). What prevents quality midwifery care? A systematic mapping of barriers in low and middle income countries from the provider perspective. PLoS One.

[64] The World Bank (2022). Classification of fragile and conflict-affected situations.

[65] Benton B, Handuleh J, Harris K, Maruthappu M, Patel P, Godman B (2014). Health in fragile states. Med Confl Survival.

[66] Newbrander W, Waldman R, Shepherd-Banigan M (2011). Rebuilding and strengthening health systems and providing basic health services in fragile states. Disasters.

[67] Beek K, McFadden A, Dawson A (2019). The role and scope of practice of midwives in humanitarian settings: a systematic review and content analysis. Hum Resour Health.

[68] Renfrew MJ, McFadden A, Bastos MH, Campbell J, Channon AA, Cheung NF (2014). Midwifery and quality care: findings from a new evidence-informed framework for maternal and newborn care. Lancet.

[69] Koblinsky M, Moyer CA, Calvert C, Campbell J, Campbell OM, Feigl AB (2016). Quality maternity care for every woman, everywhere: a call to action. Lancet.

[70] World Health Organization (2016). Standards for improving quality of maternal and newborn care in health facilities.

[71] James N, Lawson K, Acharya Y (2020). Evidence on result-based financing in maternal and child health in low-and middle-income countries: a systematic review. Glob Health Res Policy.

[72] Basinga P, Gertler PJ, Binagwaho A, Soucat AL, Sturdy J, Vermeersch CM (2011). Effect on maternal and child health services in Rwanda of payment to primary health-care providers for performance: an impact evaluation. Lancet.

