# Supplementary material for: Quality intrapartum care expectations and experiences of women in sub-Saharan African Low and Low Middle-Income Countries: a qualitative meta-synthesis
Source: BMC Pregnancy Childbirth. 2023 Jan 14;23:27. doi: 10.1186/s12884-022-05319-1 (PMC9840253; doi:10.1186/s12884-022-05319-1)
Supplement: Supplementary file 1 — Additional file 1: Supplementary file 1. Medline database search strategy. [file 12884_2022_5319_MOESM1_ESM.docx]

Supplementary file 1 Medline database search strategy

| 1 | Midwife* or midwives or (Delivery adj care) or Intra?partum or Post?partum or (Emergency adj1 obstetric*) or (Skilled adj2 attendan*) or (matern* adj care) or (maternal adj health) or Respectful or individualized or wom#n-centred or wom#n-centered or (emotional adj1 support) or (birth adj2 companion) or (effective adj1 communication) or (pain adj relief) or (referral adj plan) or (continu* adj2 care) |
| --- | --- |
| 2 | exp Delivery, Obstetric/ or exp Nurse Midwives/ or exp Midwifery/ or exp Maternal Health Services/ |
| 3 | 1 or 2 |
| 4 | expectation* or view* or perception* or prospect* or perspective* or opinion* or satisfaction* or experience* |
| 5 | exp Personal Satisfaction/ or exp Attitude/ |
| 6 | 4 or 5 |
| 7 | Angola or Benin or Burkina Faso or Burundi or Cape Verde or Cameroon or Central Republic Africa or CAR or Chad or Comoros or Cote d Ivoire or Congo or DRC or Gambia or Rwanda or Ghana or Guinea or Guinea-Bissau or Kenya or Madagascar or Malawi or Mali or Mauritania or Gambia or Rwanda or Ethiopia or Niger or Nigeria or Tanzania or Uganda or Zambia or Mozambique or Zimbabwe or Eritrea or Sao Tome or Senegal or Sierra Leone or Lesotho or Liberia or Sudan or South Sudan or Swaziland or Togo or Tanzania |
| 8 | developing countries.mp. or exp Developing Countries/ |
| 9 | exp "Africa South of the Sahara"/ |
| 10 | LLMIC* OR LMIC* |
| 11 | 7 or 8 or 9 or 10 |
| 12 | 3 and 6 and 11 |
