# Supplementary material for: Quality intrapartum care expectations and experiences of women in sub-Saharan African Low and Low Middle-Income Countries: a qualitative meta-synthesis
Source: BMC Pregnancy Childbirth. 2023 Jan 14;23:27. doi: 10.1186/s12884-022-05319-1 (PMC9840253; doi:10.1186/s12884-022-05319-1)
Supplement: Supplementary file 2 — Additional file 2: Supplementary file 2. Inclusion and exclusion criteria. [file 12884_2022_5319_MOESM2_ESM.docx]

Supplementary file 2 Inclusion and exclusion criteria

|  | Inclusion criteria | Exclusion criteria |
| --- | --- | --- |
| Population of interest | Women who used the services in health facilities during intrapartum and immediate postpartum periods. | Women who delivered at home |
| Intervention | Midwifery or intrapartum/delivery care during vaginal delivery (natural unassisted or assisted vaginal birth) provided in health facilities | Intrapartum delivery care during cesarean section was excluded as the main provider of care is unlikely to be a midwife. |
| Context | Sub-Saharan African LLMICs as defined by the World Bank. These countries are as follows: Angola, Benin, Burkina Faso, Burundi, Cape Verde, Cameroon, Central Republic Africa, Chad, Comoros, Cote d’Ivoire, DR. Congo, the Gambia, Rwanda, Ghana, Guinea, Guinea-Bissau, Kenya, Madagascar, Malawi, Mali, Mauritania, Gambia, Rwanda, Ethiopia, Niger, Nigeria, Tanzania, Uganda, Zambia, Mozambique, Zimbabwe, Eritrea, Sao Tome and Principe, Senegal, Sierra Leone, Lesotho, Liberia, Republic of Congo, Sudan, South Sudan, Swaziland, Togo, and Tanzania. | Studies from African countries located outside the Sub-Saharan Low and Low Middle-Income countries |
| Outcome of interest | Eligible studies explored one or more of the following dimensions:   - How women defined quality intrapartum services or positive childbirth experience - The expectations and experiences of women regarding the elements of ‘WHO intrapartum model’ including respectful maternity care, emotional support during childbirth, effective communication, continuity of care, skills, competency and practice of midwives and other categories of skilled birth attendants, and physical environment during childbirth. - The factors that influence a woman’s satisfaction with midwifery service during the birth of her baby and immediate postpartum care. | Studies focused on the views of healthcare providers regarding quality intrapartum care or their experiences and expectations regarding the elements of the WHO intrapartum model |
| Study design, publication type, publication date, and language | - Qualitative or mixed methods peer-reviewed primary studies conducted in Sub-Saharan African Low and Low Middle-Income countries. The qualitative data from mixed methods studies were included. - conducted between 2011 and 2021 - studies conducted in any language other than English and Arabic. - Publications of studies for which the full text can be accessed. | Any study which utilizes survey data or statistical reporting of results were excluded, as well as commentaries or discussions on the subject. |
