# Supplementary material for: Quality intrapartum care expectations and experiences of women in sub-Saharan African Low and Low Middle-Income Countries: a qualitative meta-synthesis
Source: BMC Pregnancy Childbirth. 2023 Jan 14;23:27. doi: 10.1186/s12884-022-05319-1 (PMC9840253; doi:10.1186/s12884-022-05319-1)
Supplement: Supplementary file 3 — Additional file 3: Supplementary file 3. Critical appraisal of included articles – CASP checklist. [file 12884_2022_5319_MOESM3_ESM.docx]

Supplementary file 3 Critical appraisal of included articles – CASP checklist

| **Checklist item** | Afulani et al.(2017) | Kumbani et al. (2012) | Jolly et. al (2019) | O’Donnell et al. (2014) | Maya et. al. (2018) | Namujju et. al. (2018) | Bohren et. al. (2017) | Kyaddondo et. al. (2017) | Mehretie Adinew and Abera Assefa (2017) | McMahon et. al. (2014) | Dzomeku et.al. (2017) | Oluoch-Aridi et. al. (2018) |
| --- | --- | --- | --- | --- | --- | --- | --- | --- | --- | --- | --- | --- |
| clear statement of the aims of the research | Y | Y | Y | Y | Y | Y | Y | Y | Y | Y | Y | Y |
| Is a qualitative methodology appropriate? | Y | Y | Y | Y | Y | Y | **Y** | Y | Y | **Y** | **Y** | **Y** |
| Was the research design appropriate to address the aims of the research? | Y | Y | Y | Y | Y | Y | Can't tell | Y | Y | Y | Y | Y |
| Was the recruitment strategy appropriate to the aims of the research? | Y | Y | Y | Y | Y | Y | Y | Y | Y | Can't tell | Can't tell | N |
| "Was the data collected in a way that addressed the research issue?" | Y | Y | Y | N | Y | Y | Can't tell | Y | Can't tell | Y | Y | Y |
| Has the relationship between researcher and participants been adequately considered? | N | N | N | N | N | Y | N | N | N | N | N | N |
| Have ethical issues been taken into consideration? | Y | Y | Y | Y | Y | Y | Y | Y | Y | Y | Y | Y |
| Was the data analysis sufficiently rigorous? | Y | Y | Y | Can't tell | Y | Y | Y | Y | Y | Y | Y | Y |
| Is there a clear statement of findings? | Y | Y | Y | Y | Y | Y | Y | Y | Y | Y | Y | Y |
| How valuable is the research? | Y | Y | Y | Y | Y | Y | Y | Y | Y | Y | Y | Y |
