# Supplementary material for: Quality intrapartum care expectations and experiences of women in sub-Saharan African Low and Low Middle-Income Countries: a qualitative meta-synthesis
Source: BMC Pregnancy Childbirth. 2023 Jan 14;23:27. doi: 10.1186/s12884-022-05319-1 (PMC9840253; doi:10.1186/s12884-022-05319-1)
Supplement: Supplementary file 4 — Additional file 4: Supplementary file 4. Critical appraisal of included articles – CASP checklist. [file 12884_2022_5319_MOESM4_ESM.docx]

Supplementary file 4 Critical appraisal of included articles – CASP checklist

| **Checklist item** | Kaye et. al. (2015) | Madula et. al. (2018) | Mselle et. al. (2019) | Burrowes et. al. (2017) | Bohren et. al. (2017) | Balde et. al. (2017) | Jiru and Sendo (2021) | Lavender et. al. (2021) | Ojelade et. al. (2017) | Mgawadere et. al. (2019) | Orpin et. al. (2018) | Machira and Palamuleni (2018) | Gebremichael et. al. (2018) |
| --- | --- | --- | --- | --- | --- | --- | --- | --- | --- | --- | --- | --- | --- |
| clear statement of the aims of the research | Y | Y | Y | Y | Y | Y | Y | Y | Y | Y | Y | Y | Y |
| Is a qualitative methodology appropriate? | Y | Y | Y | Y | Y | Y | Y | Y | Y | Y | Y | Y | Y |
| Was the research design appropriate to address the aims of the research? | Y | Y | Y | Y | Can’t tell | Can’t tell | Can’t tell | Y | Y | Y | Y | Y | Y |
| Was the recruitment strategy appropriate to the aims of the research? | Y | Y | Y | Y | Y | Y | Y | Y | Y | Y | Y | Y | Y |
| "Was the data collected in a way that addressed the research issue?" | Y | Y | Y | Y | Y | Y | Y | Y | Y | Y | Y | Y | Y |
| Has the relationship between researcher and participants been adequately considered? | N | N | N | Y | N | N | N | N | N | N | N | N | N |
| Have ethical issues been taken into consideration? | Y | Y | Y | Y | Y | Y | Y | Y | Y | Y | Y | Y | Y |
| Was the data analysis sufficiently rigorous? | Y | Y | Y | Y | Y | Y | Y | Y | Y | Y | Y | Y | Y |
| Is there a clear statement of findings? | Y | Y | Y | Y | Y | Y | Y | Y | Y | Y | Y | Y | Y |
| How valuable is the research? | Y | Y | Y | Y | Y | Y | Y | Y | Y | Y | Y | Y | N |
