# Supplementary material for: Quality intrapartum care expectations and experiences of women in sub-Saharan African Low and Low Middle-Income Countries: a qualitative meta-synthesis
Source: BMC Pregnancy Childbirth. 2023 Jan 14;23:27. doi: 10.1186/s12884-022-05319-1 (PMC9840253; doi:10.1186/s12884-022-05319-1)
Supplement: Supplementary file 5 — Additional file 5: Supplementary file 5. Critical appraisal of included articles – MMAT checklist. [file 12884_2022_5319_MOESM5_ESM.docx]

Supplementary file 5 Critical appraisal of included articles – MMAT checklist

| **Checklist item** | Dalinjong et. al. (2018) | Afulani et al. (2018) | Malachi et. al. (2016) | Debela et. al. (2021) | Asrese (2020) |
| --- | --- | --- | --- | --- | --- |
| S1. Are there clear research questions? | Y | Y | Y | Y | Y |
| S2. Do the collected data allow to address the research questions? | Y | Y | N | Y | Y |
| 5.1. Is there an adequate rationale for using a mixed method design to address the research question? | Y | Y | N | Y | Y |
| 5.2. Are the different components of the study effectively integrated to answer the research question? | Y | Can’t tell | can’t tell | Y | Y |
| 5.3. Are the outputs of the integration of qualitative and quantitative components adequately interpreted? | Y | Y | Y | Y | Y |
| 5.4. Are divergences and inconsistencies between quantitative and qualitative results adequately addressed? | Y | Y | can’t tell | Y | Y |
| 5.5. Do the different components of the study adhere to the quality criteria of each tradition of the methods involved? | Y | Y | N | Y | Y |
